# What’s wrong with early diagnosis of breast cancer in young women?

**DOI:** 10.3332/ecancer.2013.ed17

**Published:** 2013-03-12

**Authors:** Giovanni Codacci-Pisanelli

**Affiliations:** Universita de Roma 1, Rome, Italy

A recent paper by Johnson et al in JAMA raises a very relevant point on breast cancer in young women [Johnson et al 2013].

By analysing the huge SEER database (in its successive forms) they show that the number of young women (aged 25-39) that present with metastatic disease at the time of diagnosis has increased over the last 30 years, whereas other age groups do not show any similar increase in metastatic disease. In fact, after 1976, there was a sharp increase in localised disease, for about ten years, in older women as a consequence of increasing mammography use, but regional disease (lymph node positive) did not show any increase, and on the contrary tended to decrease.

When reading the original paper and looking closely at the figures, (as we normally do!) pay attention to the y-axis of Figures 1 and 2. The logarithmic progression tends to flatten differences occurring in the higher part of the figure. This means, for example, that the increase in distant disease for age group 40-54 is 2/100.000/year (higher than in younger patients, even if graphically the difference looks smaller) but the APC (annual percent change) is 0.61 in 40-54 and 2.07 in 25-39. Similarly, in Figure 2 the increase in incidence would look even steeper on a linear graph. The authors indicate that the increase in the 40-54 age group actually occurred before 1990.

The absolute number of young women affected is (luckily!) limited, we are talking of an increase from 1.2 to 2.6/100.000/year, but this observation is provocative and deserves further studies. Similar data for the Geneva area were published in 2007 [Bouchardy et al 2007]. At present no adequate explanation for this increase can be proposed.

We are therefore left with the impression that breast cancer still has many hidden sides and that our fight against it may require some changes. The present data may put more fuel to the debate on the use of mammography, since these young women are by definition excluded from screening.

We already mentioned one reason why only “looking” at the figures can be deceiving, but there is a further element: the y-axis of the left (localised disease) and middle part (locoregional disease) of Figure 1 go up to 400, whereas the right part (metastatic disease) only goes to 40. A tenfold difference that applies equally to all age groups. This however only refers to the situation at diagnosis, and does not include further disease evolution.

The Authors conclude that their results would acquire a stronger value if confirmed by other groups. We look forward to reading such reports and to the further evaluation of the original data, especially concerning disease evolution and mortality.
